# Predicting future weight status from measurements made in early childhood: a novel longitudinal approach applied to Millennium Cohort Study data

**DOI:** 10.1038/nutd.2016.3

**Published:** 2016-03-07

**Authors:** E Mead, A M Batterham, G Atkinson, L J Ells

**Affiliations:** 1 Health and Social Care Institute, Teesside University, Middlesbrough, UK

## Abstract

**Background/Objective:**

There are reports that childhood obesity tracks into later life. Nevertheless, some tracking statistics such as correlations do not quantify individual agreement, whereas others such as diagnostic test statistics can be difficult to translate into practice. We aimed to employ a novel analytic approach, based on ordinal logistic regression, to predict weight status of 11-year-old children from measurements at age 5 years.

**Subjects/Methods:**

The UK 1990 growth references were used to generate clinical weight status categories of 12 076 children enrolled in the Millennium Cohort Study. Using ordinal regression, we derived the predicted probability (percent chances) of 11-year-old children becoming underweight, normal weight, overweight, obese and severely obese from their weight status category at age 5 years.

**Results:**

The chances of becoming obese (including severely obese) at age 11 years were 5.7% (95% confidence interval: 5.2 to 6.2%) for a normal-weight 5-year-old child and 32.3% (29.8 to 34.8%) for an overweight 5-year-old child. An obese 5-year-old child had a 68.1% (63.8 to 72.5%) chance of remaining obese at 11 years. Severely obese 5-year-old children had a 50.3% (43.1 to 57.4%) chance of remaining severely obese. There were no substantial differences between sexes. Nondeprived obese 5-year-old boys had a lower probability of remaining obese than deprived obese boys: −21.8% (−40.4 to −3.2%). This association was not observed in obese 5-year-old girls, in whom the nondeprived group had a probability of remaining obese 7% higher (−15.2 to 29.2%). The sex difference in this interaction of deprivation and baseline weight status was therefore −28.8% (−59.3 to 1.6%).

**Conclusions:**

We have demonstrated that ordinal logistic regression can be an informative approach to predict the chances of a child changing to, or from, an unhealthy weight status. This approach is easy to interpret and could be applied to any longitudinal data set with an ordinal outcome.

## Introduction

The increasing prevalence of childhood obesity has become a major public health issue worldwide in both developing and developed countries.^1^ The consequences of childhood obesity can be severe, with an increased risk of developing conditions such as diabetes, cardiovascular disease and psychosocial disorders.^2, 3^ Furthermore, there is some evidence that children who are overweight or obese are more likely to be overweight or obese adults; hence, they are more likely to suffer from comorbidities when they reach adulthood.^4^ Nevertheless, most adults who are overweight or obese now were of normal weight as children.^5^

In England, ∼1 in 5 children aged 4–5 years and 1 in 3 children aged 10–11 years are either overweight or obese (defined using the UK90 population monitoring cut points for overweight (⩾85th centile) and obesity (⩾95th centile)). These figures are from the National Child Measurement Programme (NCMP) that was introduced into England in 2006 to measure the height and weight of children in Reception (4–5 years old) and Year 6 (10–11 years old). The rationale for introducing the NCMP included the gathering of population-level data on growth trends, informing service planning and delivery and increasing awareness of weight issues in children.^6^ The results from the programme are routinely fed back to parents via letters.^7^ There is a standard template that may be used by each local authority in England; however, some areas make changes to the letter or do not use the letter at all. This variation in practice leads to a lack of consistency in how local authorities present the results and whether they offer further support to the parents/children. In some local authorities the letter suggests that children who are overweight/obese during primary school are more likely to be overweight/obese in adulthood; some letters have previously stated that overweight or obese children are more likely to develop disorders such as cancer, diabetes and cardiovascular disease.^8^ Such information can be distressing and also confusing for parents; therefore, it is important to provide parents with information that is acceptably accurate, informative and easy to understand.

The NCMP allows the annual prevalence of childhood obesity to be reported. The NCMP also has the potential to provide prognostic information, that is, to ascertain whether an individual child is likely or not to have an unhealthy weight status when measured again later in life. Nevertheless, this issue of ‘tracking' is currently difficult to explore using NCMP data, which up until 2013 was anonymised before the annual upload to the national data collection system, thus prohibiting any data linkage on an individual level.^9^

A statistic that is used commonly in body mass index (BMI) tracking research is the correlation coefficient. In a recent meta-analysis,^10^ tracking correlations were synthesised from 48 studies that varied in their duration between initial and follow-up measurements. The authors of this review concluded that a high degree of tracking existed for follow-up durations of 1, 10 and 20 years, with respective correlation coefficients of 0.78–0.86, 0.67–0.78 and 0.27–0.47. However, a correlation coefficient does not quantify the prediction error for individual children.^11^ Odds ratios, derived from binary logistic regression models, are also commonly reported in BMI tracking research. For example, in a recent secondary analysis of the NCMP data for South Gloucestershire, England,^12^ multiple binary logistic models were used to derive over 20 separate odds ratios for boys, girls and the pooled sample across various weight categories. In this latter study, one odds ratio was cited to infer, incorrectly, that children who were overweight in Reception (85th–94th percentile, UK 1990 growth reference charts) were ‘13 times more likely' to be overweight or obese in year 6, compared with children who were between the 2nd and 49th percentile in Reception. It is not uncommon for odds ratios and relative risks to be misrepresented in research, rendering them difficult to translate to practitioners and patients.^13^ Furthermore, the analysis by Pearce *et al.*^12^ only used the population monitoring cutoffs for overweight and obesity; in the NCMP feedback letters the clinical cutoffs are used. Pearce *et al.*^12^ also did not predict the odds of a child becoming severely obese, which has shown to be an increasing concern in England.^14^ Lastly, BMI weight categories are clearly ordinal-level data, rendering the use of many binary logistic regression models across multiple pairs of weight categories non-parsimonious.

Finally, diagnostic test statistics such as sensitivity and specificity can help ascertain the individual agreement between two different measurements of status.^15^ Nevertheless, several additional statistics (for example, positive predictive value, negative predictive value and positive and negative likelihood ratios) are required for a full interpretation, rendering results that are sometimes difficult to explain to a layperson, such as a child's parent. Steurer *et al.*^16^ reported that even general practitioners can struggle to apply the statistics from the appraisal of a diagnostic test.^16^

The aim of this secondary analysis of longitudinal data was to develop a robust analytic approach to predict the individual weight status of 11-year-old-children from weight status data collected at age 5 years, and to explore the influences of sex and deprivation.

## Subjects and methods

Subjects in this secondary data analysis are from the Millennium Cohort Study (MCS) that recruited over 19 000 children born in the United Kingdom between 1 September 2000 and 11 January 2002. Children were identified from the Child Benefit register and were recruited, along with their families, when they were ∼9 months old.^17^ The study used disproportionately stratified sampling to overrepresent disadvantaged populations and areas with a high prevalence of BME (Black and Minority Ethnic) communities.^18^

Data were downloaded from the UK data archive, from sweep 1 and sweep 5 of the data collection, to select children who were of similar ages to those taking part in the NCMP (it is also possible that the children resident in England were also measured in the NCMP). The following variables were obtained: MCS research serial number, cohort member number, sex, age, BMI and index of multiple deprivation (IMD) decile (by country).^19, 20^ Height and weight were measured by study investigators at each time point, and were not self-reported. Because of the sample stratification and clustering, the data needed to be set for analysis using an attrition/nonresponse weight (whole of UK-level analysis), a finite population correction factor, a stratum variable and a ward variable to account for clustering. These variables were also obtained from the data set.^20^ As variables were required from multiple data sets, files were merged together based on the MCS research serial number and cohort member number (used to represent twins/triplets). Raw BMI values were converted into BMI *z*-scores/centiles using the LMS growth Microsoft Excel add-in^21^ where UK 1990 growth references were selected. These centiles were then converted into weight status categories using the UK 1990 clinical cutoff points: underweight (<2nd centile), normal weight (⩾2nd but <91st centile), overweight (⩾91st centile but <98th centile) and obese (⩾98th centile).^22^ These categories are also used in the NCMP feedback letters to parents.^6^ An additional category for severely obese children was also generated using the ⩾99.6th centile cutoff.^14^ IMD scores were used to assess the level of deprivation and were presented in quintiles. Ordinal logistic regression was applied to generate the predicted probability (% chances) of a child becoming underweight, normal weight, overweight, obese and severely obese at age 11 years, with weight status at age 5 years, sex, deprivation and their three-way interaction as predictors. Interaction analyses presented are exploratory. All analyses were performed using Stata software (StataCorp, 2013. Stata Statistical Software: Release 13, College Station, TX, USA: StataCorp LP). Point estimates are presented together with 95% confidence intervals. These intervals are not adjusted for multiple comparisons.^23^

Three sensitivity analyses were conducted. The first simply removed the second- and third-born twins/triplets to explore whether these had a substantial effect on the estimates. The second relaxed the constraint of the proportional odds assumption underpinning ordinal logistic regression and repeated all analyses using generalised ordinal logistic regression.^24^ This model allows the effects of the predictor variables to vary with the point at which the categories of the age 11 weight status variable are dichotomised, rather than enforcing parallel lines. Finally, we explored the effect of missing data, given that 3116 BMI values were missing at follow-up. Under a missing at random assumption, a complete case analysis—our primary analysis—is unbiased in this context and methods such as multiple imputation can only exacerbate problems by introducing additional random variation. However, multiple imputation can be used for a sensitivity analysis to examine the effects of substantial departures from the missing at random assumption. In the current study, it is plausible that those children lost to follow-up had substantially higher BMI values—that is, data missing not at random. We imputed the 3116 missing follow-up BMI values predicted from baseline BMI using the Stata ‘MI' module with predictive mean matching (random selection from 10 nearest neighbours). Twenty imputations were made by sex and deprivation strata to preserve relationships for the higher-order interactions in the analysis model. Using a pattern mixture modelling approach,^25^ each imputed follow-up BMI value was then inflated by 25% to simulate data missing not at random, with higher follow-up BMI in those not presenting for measurement at age 11 years. We then converted these inflated BMI values into weight status categories using the same method previously described. The identical ordinal logistic regression model was then applied to the 20 imputed data sets, with results combined using Rubin's rules.^26^

## Results

A total of 12 076 children were included in the analyses who had a BMI measurement along with complete data for sex and IMD score. The NCMP cleaning protocol^27^ was used to explore whether there were any BMI outliers: only two BMI measurements were slightly outside the acceptable ranges given in the protocol; hence, these were retained in the analysis. Half (50.3%) of the sample were boys, and 25.8% and 19.2% of children were in the most deprived (0 to <20%) and least deprived (80 to 100%) IMD categories, respectively. The mean BMI at baseline was 16.3±1.9 kg m^2^ and the mean age was 5.2±0.3 years. The mean BMI at follow-up was 19.2±3.7 kg m^2^ and the mean age was 11.2±0.3 years. At baseline (age 5 years), the percentage of children who were underweight, normal weight, overweight and obese (including severely obese) were as follows: 1.1% (*n=*127), 82.4% (*n=*9954), 10.3% (*n=*1249) and 6.2% (*n=*746). At follow-up (age 11 years), the percentages were as follows: 1.6% (*n=*188), 71.0% (*n=*8577), 15.1% (*n=*1819) and 12.4% (*n=*1492). The percentage of children who were severely obese at age 5 and 11 years were 2.9% (*n=*347) and 4.1% (*n=*494), respectively. The tracking of raw BMI between age 5 and age 11 years produced a correlation coefficient of 0.61.

Results from the full factorial ordinal logistic regression model are shown in Table 1, split by sex (the Stata code required to run this model is provided in Supplementary Table 1). Sex was shown to have little influence on these associations. Interestingly, overweight children had around a one-third chance of remaining overweight, one-third chance of returning to the normal-weight category and one-third chance of becoming obese. Obese (including severely obese) children at age 5 years had nearly a 70% chance of remaining obese at 11 years.

When the analysis was performed with an additional category for severe obesity, severely obese 5 year olds had a 52.8% (45.3 to 60.3%) chance of remaining severely obese at 11 years, and a 31.3% (27.4 to 35.1%) chance of decreasing their weight status and returning to the obese category (⩾98th but <99.6th centile). There were no substantial differences between sexes: severely obese boys had 49.5% (39.4 to 59.5%) chance of remaining severely obese compared with a 56.6% (46.0 to 67.2%) chance for severely obese girls. Severely obese boys and girls had a 32.3% (28.1 to 36.5%) and 30.0% (24.1 to 35.8%) chance of decreasing their weight status and becoming obese, respectively. Boys who were obese (not severe) at age 5 years had a 23.0% (17.2 to 28.8%) chance of becoming severely obese, whereas obese girls had a 27.2% (19.7 to 34.7%) chance.

Results stratified by sex and deprivation are shown in Table 2. Nondeprived obese boys had a lower chance of remaining obese at age 11 years compared with deprived obese boys; a difference of −21.8% (−40.4 to −3.2%). The opposite association was found in obese girls, where nondeprived girls were more likely to remain obese than deprived obese girls; however, this difference was not substantial. The sex difference in this specific interaction of deprivation and baseline weight status was −28.8% (−59.3 to 1.6%). No other substantial differences were found between deprived and nondeprived boys/girls or when comparing boys and girls; this was also the case when normal-weight and overweight status were predicted at follow-up (data not shown). We were unable to include underweight children in the analysis split by sex and deprivation as there were too few underweight children in the sample.

Table 3 shows the predicted percent chances of becoming severely obese by sex and deprivation. We also performed the analysis using the population monitoring cut points instead of the clinical cut points and found a slightly greater increase in the percent chances of becoming overweight or obese (results not shown). This was expected because the cut points are lower; hence, more children will have been categorised as overweight or obese.

When second- and third-born twins/triplets were removed from the analysis, there were no substantial differences in any of the predicted percent chances (data not shown). Similarly, relaxation of the constraint of the proportional odds assumption had no material effect on the findings. Results from the sensitivity analysis with missing data are shown in Table 4 for predicting obesity by sex and deprivation. When comparing the original analysis (data missing at random assumption) against the multiple imputation analysis (missing not at random assumption), no material differences were found.

## Discussion

This secondary analysis of data from the MCS has shown how a robust statistical approach can be used to predict a child's future weight status in an informative way using baseline weight status, sex and deprivation as predictor variables. This technique could be applied to NCMP data and predictions could be incorporated into the parental feedback letters, to better inform parents of the chances of their child becoming or remaining at an unhealthy weight status. In fact, this statistical technique could be applied to any longitudinal data set, and additional predictor variables could be included in the model. Furthermore, as we had a considerable proportion of missing outcome data, we have demonstrated an approach to sensitivity analysis for substantial departures from the missing at random assumption.

The main findings from the MCS analysis included showing that sex does not strongly influence the tracking of weight status from age 5 and 11 years. However, our exploratory interaction analyses suggest that deprivation might influence whether obese boys at age 5 years will remain obese at age 11 years, with nondeprived boys substantially less likely to remain obese. This association was not evident in girls. This finding is subject to replication and confirmation, but it suggests that nondeprived obese boys have a protective effect against remaining obese in later childhood, perhaps mediated by environmental and psychological factors.

Some of the children included in the MCS would have been measured in the English National Child Obesity Dataset (NCOD) in 2005–2006, which was then renamed the NCMP the following year after improvements were made.^28^ Children in the MCS would have also taken part in the NCMP in 2011–2012 when they were in year 6 of primary school. Analyses of NCMP cohort trends have shown that obesity prevalence in the most deprived children is nearly double the prevalence in the least deprived children. This inequality gap has shown to significantly increase by ∼0.5% every year, showing inequalities are continuing to widen.^29^ Analysis of cohort trends is limited because it does not explore how the weight status of individuals changes over time, and is unable to explore the influence of sex and deprivation in depth. The analysis of individual children in the MCS identified a protective effect against obesity in more affluent obese boys that would not have been seen in an analysis of cohort trends. Hence, this finding highlights the importance of obtaining linked NCMP data.

Following a change in NCMP legislation in 2013,^30^ it is now possible to upload identifiable data through an NHS number that, if submitted, will facilitate data linkage and future tracking analyses. As there are 7 years between the two measurements, the earliest any national tracking analyses could be undertaken is 2019. That said, NCMP data can be obtained locally in those areas where data have been stored on the Child Health System (CHIS), although there are lengthy and time-consuming governance procedures to overcome in order to access these data. Examples of local authorities that have obtained data via CHIS include Hull^31^ and Southampton;^32^ however, not all data were collected through the NCMP as some measurements were collected before the start of the NCMP.

The main limitation to this analysis was the large amount of missing data between baseline (age 5 years) and follow-up (age 11 years) where it was possible that these data might be missing not at random. However, we were able to conduct a sensitivity analysis that showed only small differences in predicted probabilities when data were imputed under missing not at random assumption. This finding is noteworthy, as we allowed for a large departure from the missing at random assumption, with imputed follow-up BMI values inflated by 25%. A second limitation was that some children were >5 years old at baseline and 11 years old at follow-up; however, the majority of children were close to these ages. In addition, only 1.1% of the cohort were underweight at age 5 years and only 1.6% were underweight at age 11 years. Furthermore, only 2.9% and 4.1% of children were categorised as severely obese at age 5 and age 11 years, respectively. Hence, even though we analysed over 12 000 cases, a much larger sample would be required to be able to make robust predictions using these two categories. In addition, BMI may not be the most accurate measure of a child's weight status as it has shown to not always strongly correlate with body fat distribution.^33^ However, BMI is the preferred method to use in a large sample as it is relatively quick to measure, less invasive than many other body fat assessments and has shown to be a relatively robust measurement at a population level.^34^ A final limitation of the analysis is that the majority of the sample was of white ethnicity; hence, we were unable to explore the influence of ethnicity that has shown to strongly affect the likelihood of developing obesity.^35, 36^ Furthermore, a majority of children were sampled from England; hence, we were unable to conduct a country-by-country analysis.

At present, MCS data are only freely available up age 11 years; it will be interesting to explore what effect a longer follow-up period has on predicting whether children will become overweight or obese in later life, especially as adolescence is anticipated to be an important predictor of adult weight status.^37^ In addition, it would be worthwhile to perform further analyses looking at the effect of physical activity and nutrition on changes in BMI, and also explore what factors contribute to the protective effect against obesity in nondeprived obese boys.

To conclude, this secondary data analysis has demonstrated how weight status can be tracked robustly and informatively over time. Such methods could be applied to other longitudinal data sets such as the NCMP.

## Figures and Tables

**Table 1:** The predicted percent chances of child becoming underweight, normal weight, overweight and obese at age 11 years based on their weight status at age 5 years and sex

*Weight status category and sex at age 5 years*	*Predicted percent chances of becoming each weight status category at age 11 years (95% CI)*			
	*Underweight*	*Normal weight*	*Overweight*	*Obese inc. severe*
*Underweight*				
Male	14.2 (1.9 to 26.4)	82.2 (70.6 to 93.9)	2.6 (−0.7 to 5.8)	1.0 (−0.4 to 2.4)
Female	29.9 (16.9 to 42.8)	68.2 (55.7 to 80.7)	1.4 (0.8 to 2.0)	0.5 (0.3 to 0.8)
*Normal weight*				
Male	1.4 (1.1 to 1.7)	79.7 (78.4 to 81.0)	13.0 (12.0 to 13.9)	5.9 (5.3 to 6.5)
Female	1.6 (1.3 to 1.9)	80.9 (79.7 to 82.0)	12.1 (11.3 to 12.9)	5.4 (4.9 to 6.0)
*Overweight*				
Male	0.2 (0.2 to 0.3)	38.4 (34.5 to 42.3)	31.1 (29.5 to 32.7)	30.3 (26.7 to 33.9)
Female	0.2 (0.1 to 0.2)	34.4 (30.7 to 38.0)	31.0 (29.4 to 32.6)	34.4 (31.0 to 37.9)
*Obese inc. severe*				
Male	0.0 (0.0 to 0.1)	11.8 (8.6 to 15.1)	20.6 (17.4 to 23.8)	67.6 (61.4 to 73.7)
Female	0.0 (0.0 to 0.1)	10.9 (7.8 to 14.0)	20.2 (16.3 to 24.2)	68.8 (61.9 to 75.8)

Abbreviations: CI, confidence interval; inc., including.

Numbers are rounded to 1 decimal place.

**Table 2:** The predicted percent chances of the most and least deprived children becoming obese at age 11 years based on their weight status at age 5 years and sex

*Weight status, sex and IMD (fifths) at age 5 years*		*Predicted percent chances of becoming obese (including severe) at age 11 years (95% CI)*
*Normal weight*		
Male	Most deprived (0–20%)	7.4 (6.2 to 8.6)
	Least deprived (80–100%)	4.7 (3.9 to 5.5)
Female	Most deprived (0–20%)	6.6 (5.5 to 7.7)
	Least deprived (80–100%)	3.9 (3.0 to 4.7)
*Overweight*		
Male	Most deprived (0–20%)	37.2 (29.2 to 45.3)
	Least deprived (80–100%)	27.0 (20.3 to 33.6)
Female	Most deprived (0–20%)	38.0 (30.7 to 45.3)
	Least deprived (80–100%)	30.9 (22.2 to 39.5)
*Obese inc. severe*		
Male	Most deprived (0–20%)	71.4 (61.6 to 81.2)
	Least deprived (80–100%)	49.6 (34.0 to 65.2)
Female	Most deprived (0–20%)	62.9 (50.9 to 74.9)
	Least deprived (80–100%)	69.9 (51.2 to 88.6)

Abbreviations: CI, confidence interval; IMD, index of multiple deprivation; inc., including.

Numbers are rounded to 1 decimal place.

**Table 3:** The predicted percent chances of the most and least deprived children becoming severely obese at age 11 years based on their weight status at age 5 years and sex

*Weight status, sex and IMD (fifths) at age 5 years*		*Predicted percent chances of becoming severely obese at age 11 years (95% CI)*
*Normal weight*		
Male	Most deprived (0–20%)	1.5 (1.2 to 1.8)
	Least deprived (80–100%)	0.9 (0.7 to 1.1)
Female	Most deprived (0–20%)	1.3 (1.0 to 1.6)
	Least deprived (80–100%)	0.8 (0.6 to 0.9)
*Overweight*		
Male	Most deprived (0–20%)	10.2 (7.0 to 13.5)
	Least deprived (80–100%)	6.5 (4.4 to 8.5)
Female	Most deprived (0–20%)	10.2 (7.2 to 13.1)
	Least deprived (80–100%)	7.6 (4.7 to 10.5)
*Obese (not inc. severe)*		
Male	Most deprived (0–20%)	23.8 (13.1 to 34.5)
	Least deprived (80–100%)	12.9 (6.8 to 19.0)
Female	Most deprived (0–20%)	18.9 (11.0 to 26.8)
	Least deprived (80–100%)	22.4 (11.3 to 33.4)
*Severely obese*		
Male	Most deprived (0–20%)	58.7 (41.7 to 75.7)
	Least deprived (80–100%)	32.0 (−3.7 to 67.8)
Female	Most deprived (0–20%)	46.5 (24.6 to 68.4)
	Least deprived (80–100%)	76.8 (52.7 to 100)

Abbreviations: CI, confidence interval; IMD, index of multiple deprivation; inc., including.

Numbers are rounded to 1 decimal place.

**Table 4:** Sensitivity analysis—multiple imputation of missing data showing the predicted percent chances of the most and least deprived children becoming obese at age 11 years based on their weight status at age 5 years and sex

*Weight status, sex and IMD (fifths) at age 5 years*		*Predicted percent chances of becoming obese (including severe) category at age 11 years (95% CI)*
*Normal weight*		
Male	Most deprived (0–20%)	16.3 (14.5 to 18.1)
	Least deprived (80–100%)	9.1 (7.7 to 10.5)
Female	Most deprived (0–20%)	13.1 (11.4 to 14.9)
	Least deprived (80–100%)	7.5 (6.2 to 8.8)
*Overweight*		
Male	Most deprived (0–20%)	55.5 (48.9 to 621)
	Least deprived (80–100%)	37.9 (30.6 to 45.2)
Female	Most deprived (0–20%)	53.1 (46.7 to 59.5)
	Least deprived (80–100%)	42.1 (33.1 to 51.1)
*Obese inc. severe*		
Male	Most deprived (0–20%)	82.2 (75.4 to 89.1)
	Least deprived (80–100%)	52.5 (40.3 to 64.7)
Female	Most deprived (0–20%)	74.1 (65.8 to 82.5)
	Least deprived (80–100%)	75.7 (59.3 to 92.0)

Abbreviations: CI, confidence interval; IMD, index of multiple deprivation; inc., including.

Numbers are rounded to 1 decimal place.

## References

[bib1] Ng M, Fleming T, Robinson M, Thomson B, Graetz N, Margono C et al. Global, regional, and national prevalence of overweight and obesity in children and adults during 1980–2013: a systematic analysis for the Global Burden of Disease Study 2013. Lancet 2014; 384: 766–781. 24880830 10.1016/S0140-6736(14)60460-8 PMC4624264

[bib2] Friedemann C, Heneghan C, Mahtani K, Thompson M, Perera R, Ward AM. Cardiovascular disease risk in healthy children and its association with body mass index: systematic review and meta-analysis. BMJ 2012; 345: e4759. 23015032 10.1136/bmj.e4759 PMC3458230

[bib3] Gouveia M, Frontini R, Canavarro M, Moreira H. Quality of life and psychological functioning in pediatric obesity: the role of body image dissatisfaction between girls and boys of different ages. Qual Life Res 2014; 23: 2629–2638. 24817248 10.1007/s11136-014-0711-y

[bib4] Reilly JJ, Kelly J. Long-term impact of overweight and obesity in childhood and adolescence on morbidity and premature mortality in adulthood: systematic review. Int J Obes 2011; 35: 891–898. 10.1038/ijo.2010.222 20975725

[bib5] Herman KM, Craig CL, Gauvin L, Katzmarzyk PT. Tracking of obesity and physical activity from childhood to adulthood: the Physical Activity Longitudinal Study. Int J Pediatr Obes 2009; 4: 281–288. 19922043 10.3109/17477160802596171

[bib6] HSCIC. National Child Measurement Programme: England, 2013/14 school year. December 2014 [accessed 10/08/2015]. Available from http://www.hscic.gov.uk/catalogue/PUB16070.

[bib7] Falconer CL, Park MH, Croker H, Skow Á, Black J, Saxena S et al. The benefits and harms of providing parents with weight feedback as part of the national child measurement programme: a prospective cohort study. BMC Public Health 2014; 14: 549. 24888972 10.1186/1471-2458-14-549 PMC4057922

[bib8] Statham J, Mooney A, Boddy J, Cage M. Taking stock: a rapid review of the National Child Measurement Programme. Report to the Department of Health [Internet]. April 2011 [accessed 04/05/2015]. Available from: http://eprints.ioe.ac.uk/6743/.

[bib9] Public Health England. National Child Measurement Programme: Guidance for data sharing and analysis July 2014 [accessed 10/09/2015]. Available from http://www.noo.org.uk/NCMP/analytical_guidance.

[bib10] Bayer O, Krüger H, Von Kries R, Toschke AM. Factors associated with tracking of BMI: a meta-regression analysis on BMI tracking. Obesity (Silver Spring) 2011; 19: 1069–1076. 20948517 10.1038/oby.2010.250

[bib11] Atkinson G, Nevill AM. Statistical methods for assessing measurement error (reliability) in variables relevant to sports medicine. Sports Med 1998; 26: 217–238. 9820922 10.2165/00007256-199826040-00002

[bib12] Pearce M, Webb-Phillips S, Bray I. Changes in objectively measured BMI in children aged 4-11 years: data from the National Child Measurement Programme. J Public Health e-pub ahead of print 6 May 2015; doi:10.1093/pubmed/fdv058. 10.1093/pubmed/fdv058 25948603

[bib13] Tripepi G, Jager KJ, Dekker FW, Wanner C, Zoccali C. Measures of effect: relative risks, odds ratios, risk difference, and ‘number needed to treat'. Kidney Int 2007; 72: 789–791. 17653136 10.1038/sj.ki.5002432

[bib14] Ells LJ, Hancock C, Copley VR, Mead E, Dinsdale H, Kinra S et al. Prevalence of severe childhood obesity in England: 2006-2013. Arch Dis Child 2015; 100: 631–636. 25628459 10.1136/archdischild-2014-307036

[bib15] Simmonds M, Burch J, Llewellyn A, Griffiths C, Yang H, Owen C et al. The use of measures of obesity in childhood for predicting obesity and the development of obesity-related diseases in adulthood: a systematic review and meta-analysis. Health Technol Assess 2015; 19: 1–336. 10.3310/hta19430 PMC4781104 26108433

[bib16] Steurer J, Fischer JE, Bachmann LM, Koller M, ter Riet G. Communicating accuracy of tests to general practitioners: a controlled study. BMJ 2002; 324: 824–826. 11934776 10.1136/bmj.324.7341.824 PMC100792

[bib17] Centre for Longitudinal Studies. Millennium Cohort Study. A Guide to the Datasets (Eighth Edition) First, Second, Third, Fourth and Fifth Surveys February 2014 [accessed 18/08/2015]. Available from http://www.cls.ioe.ac.uk/page.aspx?&sitesectionid=1266&sitesectiontitle=User+Guides.

[bib18] Centre for Longitudinal Studies. The Millennium Cohort Study: Technical Report on Sampling July 2007 [accessed 18/08/2015]. Available from http://www.cls.ioe.ac.uk/page.aspx?&sitesectionid=880&sitesectiontitle=Survey+Design.

[bib19] University of London Institute of Education Centre for Longitudinal Studies. Millennium Cohort Study: Third Survey, 2006 [computer file]. 6th Edition. Colchester, Essex: UK Data Archive [distributor] December 2012. SN: 5795 http://dx.doi.org/10.5255/UKDA-SN-5795-3.

[bib20] University of London Institute of Education Centre for Longitudinal Studies. Millennium Cohort Study: Fifth Survey, 2012 [computer file]. 2nd Edition. Colchester, Essex: UK Data Archive [distributor] August 2015. SN: 7464 http://dx.doi.org/10.5255/UKDA-SN-7464-2.

[bib21] LMSgrowth Microsoft Excel add-in software. Harlow Printing Limited [accessed 08/06/2015]. Available from http://www.healthforallchildren.com/shop-base/software/lmsgrowth/.

[bib22] Cole TJ, Freeman JV, Preece MA. Body mass index reference curves for the UK, 1990. Arch Dis Child 1995; 73: 25–29. 7639544 10.1136/adc.73.1.25 PMC1511150

[bib23] Rothman KJ. No adjustments are needed for multiple comparisons. Epidemiology 1990; 1: 43–46. 2081237

[bib24] Williams R. Generalized ordered logit/partial proportional odds models for ordinal dependent variables. Stata J 2006; 6: 58–82.

[bib25] Allinson P. Missing Data. Sage University Papers Series on Quantitative Applications in the Social Sciences, 07-136. Sage, 2001. pp 83–84.

[bib26] Rubin D. Multiple Imputation for Nonresponse in Surveys. Wiley: New York, 1987.

[bib27] HSCIC. Validation of National Child Measurement Programme Data. February 2015. 16/09/2015. Available from http://www.hscic.gov.uk/media/16230/Validation-of-National-Child-Measurement-Programme-Data/pdf/Validation_Principle_and_Rules.pdf.

[bib28] Public Health England. Child Obesity Data Sources [09/09/2015]. Available from https://www.noo.org.uk/data_sources/child.

[bib29] Public Health England. National Child Measurement Programme. Changes in children's BMI between 2006/7 and 2012/13 November 2014 [accessed 15/09/2015]. Available from http://www.noo.org.uk/NCMP/National_report.

[bib30] The Local Authority (Public Health, Health and Wellbeing Boards and Health Scrutiny) Regulations 2013, No. 218 (2013).

[bib31] Porter M, Greene T, Taylor A. Childhood Obesity in Hull: Paired Analysis. Hull PCT: Hull, December 2007.

[bib32] King D. Child Growth Briefing Note. National Child Measurement Programme 2001/02 to 2009/10, Summary Report October 2011 [accessed 17/08/2015]. Available from http://www.publichealth.southampton.gov.uk/Images/Child%20Growth%20Report%20SC%20PCT%20(2011).pdf.

[bib33] Javed A, Jumean M, Murad MH, Okorodudu D, Kumar S, Somers VK et al. Diagnostic performance of body mass index to identify obesity as defined by body adiposity in children and adolescents: a systematic review and meta-analysis. Pediatr Obes 2015; 10: 234–244. 24961794 10.1111/ijpo.242

[bib34] Dinsdale H, Ridler C, Ells LJ. A simple guide to classifying body mass index in children. Oxford: National Obesity Observatory, 2011. Available from http://www.noo.org.uk/uploads/doc/vid_11601_A_simple_guide_to_classifying_BMI_in_children.pdf.

[bib35] Freedman DS, Khan LK, Serdula MK, Dietz WH, Srinivasan SR, Berenson GS. Racial differences in the tracking of childhood BMI to adulthood. Obes Res 2005; 13: 928–935. 15919847 10.1038/oby.2005.107

[bib36] Karlsen S, Morris S, Kinra S, Vallejo-Torres L, Viner RM. Ethnic variations in overweight and obesity among children over time: findings from analyses of the Health Surveys for England 1998-2009. Pediatr Obes 2014; 9: 186–196. 23554401 10.1111/j.2047-6310.2013.00159.x PMC4171811

[bib37] Dietz WH. Critical periods in childhood for the development of obesity. Am J Clin Nutr 1994; 59: 955–959. 8172099 10.1093/ajcn/59.5.955

