# Supplementary material for: Predicting future weight status from measurements made in early childhood: a novel longitudinal approach applied to Millennium Cohort Study data
Source: Nutr Diabetes. 2016 Mar 7;6(3):e200–. doi: 10.1038/nutd.2016.3 (PMC4817076; doi:10.1038/nutd.2016.3)
Supplement: Supplementary Table 1 [file nutd20163x1.docx]

| **Action** | **Stata® software code** |
| --- | --- |
| Due to the complex survey design in the MCS, we used a design-based approach to set the data for analysis using a sampling/ attrition weight for a whole of UK-level analysis (*EOVWT2*), a Finite Population Correction factor (*NH2*), a stratum variable (*PTTYPE2*), and electoral ward as the primary sampling unit (*SPTN00*) | *svyset SPTN00 [pweight=EOVWT2], strata(PTTYPE2) fpc(NH2)* |
| The ordinal logistic regression command can then be run using the weight category at age 11 as the outcome variable (*clinweightcat11*) and the predictor variables as main effects and interactions: weight category at age 5 (*clinweightcat5*), sex and IMD fifths (*IMD_quin_age5*). Also “if *EOVWT2>-1*” is added as some missing values were coded as -1. | *svy: ologit clinweightcat11 i.clinweightcat5##i.sex##i.IMD_quin_age5 if EOVWT2>-1* |
| The results can then by split by any one of the predictor variables. The code includes predicting underweight (0), normal weight (1), overweight (2) and obese (3) at age 11, and the following code can be used to split the results by age 5 weight status only: | *margins, at (clinweightcat5 =(0/3)) predict(outcome(0))*  *margins, at (clinweightcat5 =(0/3)) predict(outcome(1))*  *margins, at (clinweightcat5 =(0/3)) predict(outcome(2))*  *margins, at (clinweightcat5 =(0/3)) predict(outcome(3))* |
| A code can then be used to also split the results by sex. | *margins sex, at (clinweightcat5 =(0/3)) predict(outcome(0))*  *margins sex, at (clinweightcat5 =(0/3)) predict(outcome(1))*  *margins sex, at (clinweightcat5 =(0/3)) predict(outcome(2))*  *margins sex, at (clinweightcat5 =(0/3)) predict(outcome(3))* |
| Results can then also be split by sex and deprivation using the following codes (underweight children are not included due to sparse data): | *margins sex#IMD_quin_age5, at (clinweightcat5 =(1/3)) predict(outcome(1))*  *margins sex#IMD_quin_age5, at (clinweightcat5 =(1/3)) predict(outcome(2))*  *margins sex#IMD_quin_age5, at (clinweightcat5 =(1/3)) predict(outcome(3))* |

| After running the above analysis, the following code produces the comparisons between different levels of deprivation by sex for each weight status category at age 5. For example, from the Results section of the manuscript: “Non-deprived obese boys had a lower chance of remaining obese at age 11 compared to deprived obese boys; a difference of -21.8% (-40.4% to -3.2%).” This effect is given by the probability of a non-deprived obese 5-year old boy remaining obese at age 11 (49.6%) minus the probability of a deprived obese 5-year old boy remaining obese at age 11 (71.4%) – see Table 2. | *margins r.IMD_quin_age5@sex, at (clinweightcat5=(1/3)) predict(outcome(1))*  *margins r.IMD_quin_age5@sex, at (clinweightcat5=(1/3)) predict(outcome(2))*  *margins r.IMD_quin_age5@sex, at (clinweightcat5=(1/3)) predict(outcome(3))* |
| --- | --- |
| The following code then produces the estimate of the difference between boys and girls for the deprivation by baseline weight status interaction. For example, from the Results: “The sex difference in this specific interaction of deprivation and baseline weight status was  -28.8% (-59.3% to 1.6%).” This effect is given by the probability of remaining obese at age 11 in obese (age 5) non-deprived vs. deprived boys (-21.8%, as above) minus the same comparison in girls (7.0%. See Table 2: 69.9% minus 62.9%). | *margins r.IMD_quin_age5#r.sex, at (clinweightcat5=(1/3)) predict(outcome(3))* |
| The same analysis can also be done with an extra column for severe obesity; however, it is important to remember that the “obese” category will now only represent children between the 91^st^ and 99.6^th^ centiles. | *svyset SPTN00 [pweight=EOVWT2], strata(PTTYPE2) fpc(NH2)*  *svy: ologit clincat_11_SEV i.clincat_5_SEV##i.sex##i.IMD_quin_age5*  *margins, at (clincat_5_SEV =(0/4)) predict(outcome(4))*  *margins sex, at (clincat_5_SEV =(0/4)) predict(outcome(4))*  *margins sex# IMD_quin_age5, at (clincat_5_SEV =(1/4))predict(outcome(4))*  *Etc.* |

**Table S1. Stata® software code required to perform the ordinal logistic regression analysis presented in the manuscript**
